# The Whitening Efficacy of a Hydroxyapatite Toothpaste and a Blue Covarine Toothpaste: A Comparative In Vitro Study

**DOI:** 10.3390/dj13040143

**Published:** 2025-03-26

**Authors:** Joachim Enax, Pascal Fandrich, Erik Schulze zur Wiesche, Bennett T. Amaechi

**Affiliations:** 1 Research Department, Dr. Kurt Wolff GmbH & Co. KG, 33611 Bielefeld, Germany; pascal.fandrich@drwolffgroup.com (P.F.); erik.schulzezurwiesche@drwolffgroup.com (E.S.z.W.); 2 Department of Comprehensive Dentistry, School of Dentistry, University of Texas Health San Antonio, San Antonio, TX 78229-3900, USA; amaechi@uthscsa.edu

**Keywords:** teeth, tooth whitening, stain removal, toothpaste, hydroxyapatite, blue covarine

## Abstract

**Background**: There is high demand for white and healthy teeth. Therefore, various whitening toothpastes are commercially available. Hydroxyapatite and blue covarine are two ingredients used in whitening toothpaste formulations. **Objectives**: This in vitro study analyzed the stain-removing efficacy of two commercial whitening toothpastes: one containing hydroxyapatite and the other containing blue covarine. **Methods**: The stain-removing efficacy of both toothpastes was analyzed for different brushing times (30 and 180 s) using a staining model on human enamel. Photographic documentation and colorimetric measurements were performed after staining and after each brushing series. Colorimetric measurements were used to determine the stain removal efficacy in percentage. Statistical analysis was performed using one-way analysis of variance (ANOVA) with a post hoc Tukey test and Levene’s test to assess the homogeneity of variances. The significance level α was set at 0.05. **Results**: The hydroxyapatite toothpaste demonstrated a significantly higher stain-removing efficacy compared to the blue covarine toothpaste after 30 s of brushing. The stain-removing efficacy was comparable between the two toothpastes after 180 s of brushing. **Conclusions**: Both hydroxyapatite and blue covarine toothpastes effectively cleaned stained enamel, with hydroxyapatite toothpaste showing a significantly higher stain-removing efficacy after 30 s of brushing.

## 1. Introduction

Esthetic dentistry includes various procedures, many of which can only be performed in a dental office. In addition to various patient needs concerning esthetic dentistry, there is a particularly high demand for white and healthy teeth [1,2,3]. Various factors, such as exogenous stains from coffee, tea, smoking, etc., can negatively affect the color of teeth. Additionally, a person’s age and dietary habits can have an influence on the tooth color since dentin become more visible as enamel is lost [4]. One prominent procedure in esthetic dentistry is tooth bleaching, which uses high concentrations of hydrogen peroxide or carbamide peroxide to whiten teeth [3]. However, certain side effects, such as sensitivity, may occur after in-office bleaching [3]. During daily oral care, patients can contribute to whiter teeth by using whitening toothpastes [1].

In general, toothpastes contain various ingredients [5]. Additionally, certain toothpastes are specially formulated for tooth whitening [1,5].

Whitening agents in toothpastes include various ingredients [1]. In addition to the composition of the toothpaste, the quantity of the toothpaste dispensed onto the toothbrush significantly influences the whitening efficacy. It has been shown in an in vitro study that larger toothpaste amounts (full length of brush) remove stains much more efficiently than smaller toothpaste amounts (pea-sized or grain-of-rice-sized amounts) [6].

The whitening effect of many toothpastes is mainly based on their abrasive systems, which in most cases are hydrated silica [5]. As mentioned previously, whitening toothpastes can include a range of ingredients beyond abrasives. Hydroxyapatite [7,8,9,10,11] and blue covarine [1,12,13,14,15] are two examples of toothpaste ingredients that induce an optical whitening effect. Both ingredients are used in whitening toothpastes worldwide (Table 1).

Hydroxyapatite, Ca_5_(PO_4_)_3_(OH), is an inorganic agent in oral care products and has a whitish appearance. Hydroxyapatite-based toothpastes have been shown to be effective in caries prevention [16] and in reducing the symptoms of dentin hypersensitivity [17]. Their whitening effects have been shown in vitro and in vivo in various studies, as reviewed by Limeback et al. [7]. A main advantage of hydroxyapatite is its high biocompatibility, making it a safe and efficient active ingredient for all age groups [18].

Blue covarine, C_32_H_16_CuN_8_, exhibits a blue appearance and it is a frequently used colorant in commercial whitening toothpastes [1,12,14,15]. Its whitening effect is achieved by shifting the color absorption and reflection spectra from yellow to blue [1,15]. Blue covarine is chemically copper phthalocyanine, i.e., an organic copper complex [19]. The whitening effect of toothpastes containing blue covarine has been demonstrated in in vitro studies and in clinical studies [1,12,13,14].

In vitro models that analyze the whitening efficacy of toothpastes using stained tooth samples and brushing them under defined conditions are widely used in dental research [6,13,14,20,21]. Stookey et al. found that the results of these in vitro tests can correlate with the stain-removing effects observed in vivo [20]. While the whitening effect of toothpastes is often described as stain-removing efficacy from tooth surfaces only (e.g., as assessed by the pellicle cleaning ratio (PCR) [22]), there are active ingredients in toothpastes that produce whitening effects beyond stain removal [1].

Both hydroxyapatite [7,8,9] and blue covarine [1,12,13,14,15] are used as whitening agents in commercial whitening toothpastes (Table 1). However, to date, a direct head-to-head comparison between a commercial hydroxyapatite-based toothpaste and a commercial toothpaste with blue covarine is lacking. Thus, the aim of this in vitro study was to compare the stain-removing efficacy of two commercially available toothpastes, i.e., a hydroxyapatite-based toothpaste and a toothpaste containing blue covarine.

## 2. Materials and Methods

### 2.1. Toothpastes

The stain-removing efficacy of two commercial whitening toothpastes was tested using an in vitro model [6]. The compositions of the toothpastes are summarized in Table 2.

Both toothpastes were analyzed in a blinded manner, meaning they were dispensed into neutral toothpaste tubes that only differed by their blinding codes. Unblinding was performed after the statistical analysis.

### 2.2. Experimental Protocol

This study was performed at the Fraunhofer Institute for Microstructure and Systems IMWS, Halle (Saale), Germany. Human teeth were received from Indiana University School of Dentistry, Oral Health Research Institute. Enamel samples were prepared from extracted, intact human molars (caries-free, collection anonymized). The teeth were disinfected in a 10% hydrogen peroxide solution for 48 h, then rinsed with water, and subsequently stored in an ethanol–thymol mixture until use. These specimens were embedded in epoxy resin and ground to a 1200-grit finish. The samples underwent a mild etching process by immersion in a 1% hydrochloric acid solution for one minute, and were exposed afterward to a saturated sodium carbonate solution for two minutes, and then to a 1% phytic acid solution for one minute [22]. Human molars were separated into specimens with a diamond-bladed saw. Two specimens are prepared from each tooth crown, embedded in epoxy resin (EpoFix, Struers, Ø14 mm), and ground stepwise from 800 grit up to 1200 grit (Presi-Minitech-333, polishing machine).

The enamel staining procedure was performed according to a modified protocol from Lath et al. [23]. The enamel samples were rinsed with artificial saliva for two minutes at room temperature, followed by deionized water. They were then exposed to a chlorhexidine-containing mouth rinse (Chlorhexamed Forte 0.2%, GSK) for two minutes at room temperature and rinsed again with deionized water. Subsequently, the enamel samples were immersed in black tea for 60 min at 37 °C, followed by a rinsing step and air drying. This process was repeated for a total of four cycles.

The tea was prepared by infusing one Typhoo one-cup tea bag in 50 mL of boiling water in a Duran bottle and stirring at room temperature for 5 min using a magnetic stirrer. After 5 min, the tea bag was removed. The artificial saliva was prepared according to a formulation described by Pratten et al. [24].

The tests were conducted using the TePe Select™ medium toothbrush. Prior to brushing, the toothbrushes were moistened with deionized water under standardized conditions. The enamel samples were brushed with a toothpaste slurry at a 1:1 *w*/*w* dilution ratio (25 g of toothpaste:25 g of deionized water) [6]. The brushing procedure was carried out using a mechanical brushing device (V8 brushing simulator, JWE GmbH). During this process, the toothbrushes were operated in a reciprocating (linear) motion across the surfaces of the enamel samples using a brushing force of 1.5 N and a brushing frequency of 2.5 Hz. Brushing was performed for 30 and 180 s using a slurry volume of 50 mL per sample. The enamel samples were meticulously rinsed with deionized water following the brushing procedure. To obtain representative results, a total of six enamel samples per group were tested.

### 2.3. Analysis

Images of human enamel surfaces were captured under standardized conditions using a reflex camera (Canon EOS 600D with a 55 mm focal length; Program M, ISO 200, aperture 5.6, shutter speed 1/30, high quality of the photos 3456 × 5184 pics: jpeg format, black background). The room lighting and sample positioning were standardized. The reflection mode output was measured in L*a*b*-color values. The evaluation was conducted using SpectraMagic Software by Konica Minolta.

The stain removal performance was determined using L*a*b* colorimetric measurements (spectrophotometer CM-3600A, Konica Minolta), which were performed at baseline, after staining, and after stain removal on the same measurement area with a diameter of 4 mm. During the measurements, it was ensured that the samples were positioned in a standardized way by making marks on the sample. The stain removal performance (expressed as a percentage) was calculated using the formula [(Δ*E*_1_/Δ*E*_2_) × 100%], where Δ*E*_1_ represents the colorimetric difference between the cleaned and stained tooth surfaces, and Δ*E*_2_ represents the colorimetric difference between the baseline tooth surface and stained tooth surface. The formulas for calculating Δ*E*_1_ and Δ*E*_2_ can be found in a previous publication [6]. The mean and standard deviation of 6 individual measurements per toothpaste group were calculated.

Statistical analyses were carried out using one-way analysis of variance (ANOVA) with a post hoc Tukey test and Levene’s test to assess the homogeneity of variances (Origin23b, OriginLab Corporation, Northampton, MA, USA). The significance level α was set at 0.05.

## 3. Results

**Figure 1:**
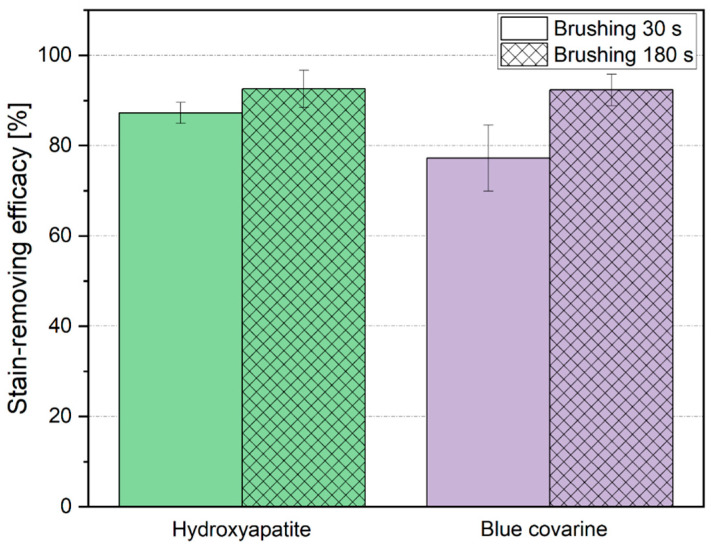
In vitro stain-removing efficacy of the hydroxyapatite toothpaste and the blue covarine toothpaste on enamel after 30 s and 180 s of tooth brushing.

**Figure 2:**
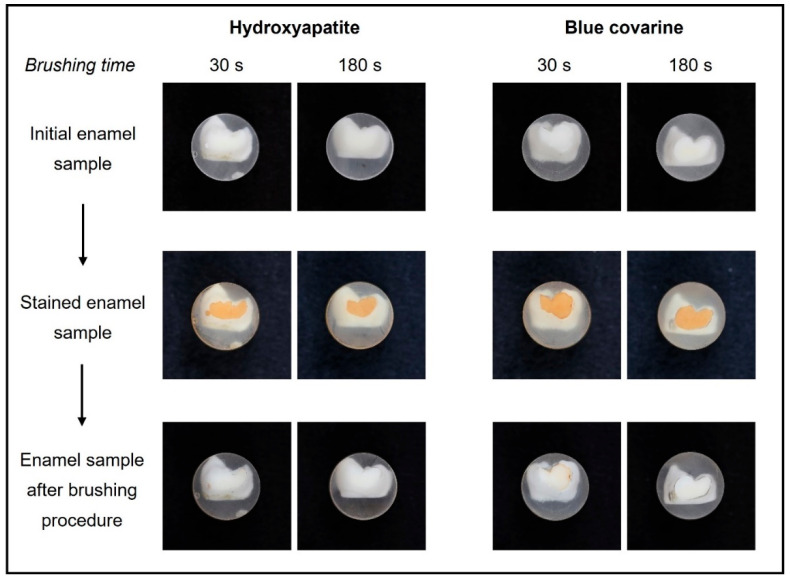
Representative photographs showing the in vitro stain-removing efficacy of the hydroxyapatite toothpaste and the blue covarine toothpaste on enamel after 30 s and 180 s tooth brushing.

The results of the in vitro stain-removing efficacy of the hydroxyapatite toothpaste and the blue covarine toothpaste after 30 s and 180 s of tooth brushing are summarized in Table 3, as well as Figure 1. Representative photographs showing the stain-removing efficacy of the tested toothpastes are depicted in Figure 2.

After 30 s of brushing, the hydroxyapatite toothpaste (stain-removing efficacy: 87.3 ± 2.1%) was significantly more effective at stain removal on enamel than the blue covarine toothpaste (*p* < 0.01, Table 3). After 180 s of brushing, there was no significant difference in the stain-removing efficacy between the two types of toothpaste (*p* = 0.99, Table 3). The stain-removing efficacy significantly improved when the brushing time was increased from 30 s to 180 s for both the hydroxyapatite toothpaste (*p* < 0.05) and the blue covarine toothpaste (*p* < 0.01, Table 3).

The stain-removing efficacy of hydroxyapatite toothpaste and blue covarine toothpaste is depicted in the photographs presented in Figure 2. The intense brownish staining was removed by the hydroxyapatite toothpaste after brushing for 30 s and 180 s, resulting in white enamel surfaces (Figure 2). The blue covarine toothpaste also led to a visible stain-removing effect; however, after 30 s, some remaining brownish stains were still visible on the enamel surfaces (Figure 2).

## 4. Discussion

There is a high demand for white teeth, and whitening toothpaste can help consumers achieve whiter teeth [1]. Two commercial whitening toothpastes were analyzed for their in vitro stain-removing efficacy: a hydroxyapatite toothpaste and a blue covarine toothpaste. Both hydroxyapatite [7,8,9,10,11] and blue covarine [1,12,13,14,15] are active ingredients used in whitening toothpastes (see also Table 1). The in vitro model used in the present study has been described in the literature [6]. Furthermore, similar in vitro models have previously been established in the dental literature [20,21].

The tested toothpastes can be used safely on a daily basis because they are free from abrasives with high relative hardness, such as alumina and perlite (which are both harder than hydrated silica), indicating that the tested toothpastes are gentle on teeth, particularly on exposed dentin and gingiva. The whitening effect of both toothpastes can be explained by their abrasive system (hydrated silica) as well as by special whitening ingredients [1]. The hydroxyapatite toothpaste contains hydroxyapatite, while the blue covarine toothpaste contains blue covarine, which is chemically known as copper phthalocyanine. The blue covarine toothpaste contains an additional pigment, CI 74260 (phthalocyanine green G), which is a green pigment.

The stain-removing effects of toothpastes containing blue covarine have been demonstrated in in vitro studies [13,14]. However, due to differences in the experimental setup (e.g., slightly different tooth staining protocols), a direct comparison with the results of the present study may not be possible. Blue covarine leads to the impression of whiter teeth by altering the optical properties of enamel when deposited on the tooth surface [15,25,26,27]. On the other hand, some studies question the whitening effect of blue covarine toothpastes under in vivo conditions [28,29,30].

Although marketed as whitening toothpastes, both products serve as ‘regular’ toothpastes, used 2–3 times daily because they also contain caries-preventive active ingredients, i.e., 20% hydroxyapatite (hydroxyapatite toothpaste) [31] and 1450 ppm fluoride as sodium fluoride (blue covarine toothpaste) [32].

After 30 s of tooth brushing, the stain-removing efficacy of hydroxyapatite toothpaste was significantly higher (87.3 ± 2.1%) than that of blue covarine toothpaste (77.2 ± 7.3%) (Table 3). However, after 180 s of tooth brushing, the stain-removing efficacies of both toothpastes were almost identical and were approximately 92%, indicating that almost all stains have been removed after this intensive brushing. If consumers brush their teeth for 3 min (180 s) and consider a maximum of 128 tooth surfaces in the dentition of healthy adults, it can be calculated that each tooth surface is cleaned for about 1.4 s. This calculation has the limitations that under real-life conditions the toothbrush does not brush only one surface at a time and that not all tooth surfaces are brushed equally, but the calculation provides a rough overview. Using this estimation, brushing for 30 s on one tooth surface means that a cumulative of approximately 21 tooth brushing episodes were performed, and brushing for 180 s means that a cumulative of approximately 128 tooth brushings were performed. The results obtained for 30 s of tooth brushing suggest that after a relatively short period, stains can be removed efficiently with both toothpastes. However, the chosen in vitro conditions do not include the adhesion of new stains to the enamel surface, which is a limitation, especially for the 180 s tooth brushing procedure (approximately a cumulative of 128 tooth brushings).

The color of teeth is not solely caused by exogenous stains from coffee, tea, smoking, etc., but it is also related to the individual’s age [4]. It is known that enamel wear leads to the underlying dentin becoming more visible, which, in turn, makes the tooth appear darker [4]. Therefore, it is crucial to avoid using highly abrasive whitening toothpastes. Hydroxyapatite can also help to counteract enamel wear as it remineralizes the enamel [33] and forms a protective layer [34].

The 20% hydroxyapatite in hydroxyapatite toothpaste is beneficial since it has been demonstrated that there is a correlation between the hydroxyapatite concentration in toothpastes and its whitening efficacy. For example, Niwa et al. investigated the whitening and brightening efficacy of toothpastes without and with hydroxyapatite (3% and 15%) and reported that both whiteness and brightness increased with higher hydroxyapatite concentrations in the toothpastes [8]. This is supported by a study of Shang et al., which demonstrated that a toothpaste containing 10% hydroxyapatite led to a greater whitening effect than a toothpaste with 1% hydroxyapatite [10].

The effectiveness of certain whitening ingredients can also depend on their ability to adhere to tooth surfaces. To the best of the authors’ knowledge, no studies have been published that directly compare the adhesion mechanisms of hydroxyapatite and blue covarine on enamel surfaces. However, hydroxyapatite is known to interact with enamel surfaces [34,35]. This interaction can be attributed to the fact that the hydroxyapatite used in tested toothpastes is of similar nature (structure and composition) to the crystallites found in human enamel. Furthermore, hydroxyapatite not only forms a whitening layer on the enamel [34], but also contributes to the remineralization of demineralized enamel surfaces [33], which supports tooth surface whitening by masking the dentin color that becomes increasingly visible as the enamel is worn down with age [7]. In addition to tooth whitening, hydroxyapatite toothpastes are known to prevent caries [31], reduce symptoms of dentin hypersensitivity [17], and improve gingival health [36]. Hydroxyapatite exhibits various mechanisms of action in preventive oral healthcare [31].

The use of the two commercial toothpastes in the present study has advantages but also limitations. The toothpastes tested are readily available products, meaning they are the exact formulations a consumer would use for daily oral care. However, this also presents a limitation for a direct comparison of the whitening agents. The observed whitening efficacy may not be solely attributed to the primary whitening agents (hydroxyapatite vs. blue covarine), instead, other ingredients within the complex toothpaste formulations may have had an influence on the results. This is a limitation of this study. The main ingredients, which may have influenced the stain-removing efficacy of the toothpastes will be discussed as follows: Both toothpastes contain hydrated silica, which is known to effectively remove plaque and stains [5]. Tetrapotassium pyrophosphate in the hydroxyapatite toothpaste may have influenced the whitening effect because pyrophosphates are used as anti-calculus agents and abrasives in toothpastes, and they are also known for supporting the desorption of stains from the enamel surface [1,5]. The tooth stain-removing effect of the blue covarine toothpaste may have been influenced by the presence of sodium lauryl sulfate, which is a potent foaming agent (surfactant). Surfactants are known to remove hydrophobic compounds (including stains) from the tooth surface.

Furthermore, even with a similar abrasive system, such as hydrated silica (present in both the hydroxyapatite toothpaste and the blue covarine toothpaste), the quality and quantity of the abrasive particles may affect the toothpaste’s cleaning and, consequently, its whitening effectiveness.

The present study focused on the stain-removing effect of two different toothpastes, but no control group without toothpaste was included; thus, the isolated influence of the toothbrush alone on stain removal was not analyzed. This is a limitation of the study. The stain-removing effect of the toothbrush alone could be performed in future studies. However, under real-life conditions, it would be unusual for a consumer to brush his teeth without toothpaste, and at least in terms of abrasive potential, the impact of the toothbrush alone appears small compared to the combination of toothpaste and toothbrush together [37]. Additionally, a study from Wang et al. found that brushing with tap water only (i.e., without toothpaste) does not lead to a significant stain-removing effect under in vitro conditions [21].

The Relative Dentin Abrasivity (RDA) values of both toothpastes were not analyzed in this study. However, it has been reported that similar toothpastes containing hydrated silica as the main abrasive typically have an RDA range of approximately 60–90 [38].

Future research could involve comparing two experimental toothpastes that have the same base formulation, including the same abrasive system (i.e., the same RDA value), the same surfactant system, etc., but differ only in their active ingredients (hydroxyapatite vs. blue covarine). Moreover, clinical studies could be performed to further substantiate the results of the present study. This study focuses on immediate stain removal but does not address the durability of whitening effects. Thus, the durability of the whitening effect of both toothpastes should be analyzed in future studies. In addition to the two toothpastes tested, future studies could also evaluate other toothpastes, such as those containing different types of abrasives or other whitening agents.

## 5. Conclusions

The tested hydroxyapatite-based toothpaste demonstrated effectiveness in reducing stains on enamel. The stain-removing efficacy of the hydroxyapatite-based toothpaste was significantly higher than that of the tested blue covarine toothpaste after 30 s of brushing on an enamel surface (a cumulative of approximately 21 brushing episodes).

## Figures and Tables

**Table 1:** Overview of the results of a global product search conducted using the Mintel database, specifically targeting whitening toothpastes containing hydroxyapatite and blue covarine (as examples of toothpaste ingredients that produce an optical whitening effect), as well as hydrogen peroxide (for comparison).

Toothpaste with Whitening Claims	Number of Whitening Toothpastes	Ingredient Percentage (%)
Blue covarine ^a^	250	17.3
Hydroxyapatite ^b^	106	7.3
Hydrogen peroxide ^c^	39	2.7
Total number	1443	---

Only toothpastes released between May 2023 and May 2024 were included. The search terms provided by the Mintel database were ^a^: CI 74160; ^b^: hydroxyapatite OR calcium hydroxyapatite OR zinc hydroxyapatite OR calcium/magnesium/zinc hydroxyapatite; ^c^: hydrogen peroxide. Note that whitening toothpastes may contain one or more whitening agents. Some whitening toothpastes may contain only abrasives.

**Table 2:** Compositions of the tested toothpastes.

Hydroxyapatite toothpaste (Bioniq^®^ Repair Whitening Toothpaste, Dr. Kurt Wolff GmbH & Co. KG, Germany)	Blue covarine toothpaste (Signal White Now Toothpaste, Unilever, The Netherlands)
Aqua, Hydroxyapatite (20%), Glycerin, Hydrated Silica, Sorbitol, Sodium Myristoyl Sar-cosinate, Tetrapotassium Pyrophosphate, Sodium Methyl Cocoyl Taurate, Aroma, Cellulose Gum, Propanediol, Silica, Zinc PCA, Sodium Saccharin, Sodium Chloride, Phenoxyethanol, Menthol, Mentha Oiperita Oil, Anethole, Limonene, Pinene, Be-ta-caryophyllene, Terpineol, Carvone.	Hydrogenated Starch Hydrolysate, Aqua, Hydrated Silica, Sodium Lauryl Sulfate, Aroma, Cellulose Gum, Sodium Fluoride, Sodium Saccharin, PVM/MA Copolymer, Glycerin, Trisodium Phosphate, Limonene, CI 74160, CI 74260. (Contains sodium fluoride [1450 ppm fluoride]; CI 74160: Blue covarine, i.e., copper phthalocyanine (C_32_H_16_CuN_8_)).

**Table 3:** In vitro stain-removing efficacy of the hydroxyapatite toothpaste and the blue covarine toothpaste on enamel after 30 s and 180 s of tooth brushing (for graphical visualization of the results see Figure 1).

	Mean Stain-Removing Efficacy in % (±Standard Deviation)	
Toothpaste	30 s Brushing	180 s Brushing
Hydroxyapatite toothpaste	87.3 ± 2.1	92.6 ± 4.1
Blue covarine toothpaste	77.2 ± 7.3	92.4 ± 3.5

## Data Availability

Data are contained within the article.
